# Deep Sequencing of the T-cell Receptor Repertoire Demonstrates Polyclonal T-cell Infiltrates in Psoriasis

**DOI:** 10.12688/f1000research.6756.1

**Published:** 2015-08-03

**Authors:** Jamie L. Harden, David Hamm, Nicholas Gulati, Michelle A. Lowes, James G. Krueger

**Affiliations:** 1 Laboratory for Investigative Dermatology, The Rockefeller University, New York, NY, USA; 2 Adaptive Biotechnologies, Seattle, WA, USA; 3 Division of Dermatology, Montefiore Medical Center, Albert Einstein College of Medicine, Bronx, NY, USA

**Keywords:** T-cell receptor (TCR), TCR deep-sequencing, psoriasis, T-cell, polyclonal, αβ-T-cells, γδ- T-cells

## Abstract

It is well known that infiltration of pathogenic T-cells plays an important role in psoriasis pathogenesis. However, the antigen specificity of these activated T-cells is relatively unknown. Previous studies using T-cell receptor polymerase chain reaction technology (TCR-PCR) have suggested there are expanded T-cell receptor (TCR) clones in psoriatic skin, suggesting a response to an unknown psoriatic antigen. Here we describe the results of high-throughput deep sequencing of the entire αβ- and γδ- TCR repertoire in normal healthy skin and psoriatic lesional and non-lesional skin. From this study, we were able to determine that there is a significant increase in the abundance of unique β- and γ- TCR sequences in psoriatic lesional skin compared to non-lesional and normal skin, and that the entire T-cell repertoire in psoriasis is polyclonal, with similar diversity to normal and non-lesional skin. Comparison of the αβ- and γδ- TCR repertoire in paired non-lesional and lesional samples showed many common clones within a patient, and these close were often equally abundant in non-lesional and lesional skin, again suggesting a diverse T-cell repertoire. Although there were similar (and low) amounts of shared β-chain sequences between different patient samples, there was significantly increased sequence sharing of the γ-chain in psoriatic skin from different individuals compared to those without psoriasis. This suggests that although the T-cell response in psoriasis is highly polyclonal, particular γδ- T-cell subsets may be associated with this disease. Overall, our findings present the feasibility of this technology to determine the entire αβ- and γδ- T-cell repertoire in skin, and that psoriasis contains polyclonal and diverse αβ- and γδ- T-cell populations.

## Introduction

Psoriasis vulgaris (plaque psoriasis) is a chronic, inflammatory skin disease characterized by raised, red scaly plaques ( Lowes *et al.*, 2014). It has been well established that infiltration of pathogenic T-cells plays an important role in disease pathology, particularly T-cells with the ability to produce interleukin (IL)-17 and IFN-γ ( Lowes *et al.*, 2014; Perera *et al.*, 2012). T-cell targeting and specifically biologics targeting the T _H_17 axis, have proven to be extremely efficacious in treatment of psoriasis ( Lowes *et al.*, 2014). However, despite the established role of T-cells in psoriasis pathogenesis, the potential auto-antigens eliciting disease or the antigen specificity of T-cells contributing to psoriasis are relatively unknown.

High-throughput sequencing of the T-cell receptor (TCR) repertoire can help elucidate whether a narrow subset of T-cells have undergone clonal expansion, potentially elicited by a particular antigen. Previous evaluation of TCR repertoire in psoriasis has shown preferential usage of particular β-chain variable gene segments ( Chang *et al.*, 1995; Menssen *et al.*, 1995). However in these studies, a small number of TCR sequences was obtained from a potentially vast underlying diversity of TCR sequences ( Robins *et al.*, 2009) and clonal expansion was determined only in the context of particular Vβ-chain usages. Recent advances in next generation deep sequencing have enabled the quantitative sampling of a significantly larger enough fraction of the T-cell repertoire to make strong inferences regarding the diversity of the T-cell repertoire and measure the degree of oligoclonality based on individual clone lineages ( Robins, 2013).

Here we present the first exploration of TCR deep sequencing in psoriatic (both lesional and non-lesional) and normal skin using the ImmunoSEQ assay (Adaptive Biotechnologies, Seattle, WA, USA). Genomic DNA (gDNA) and complementary DNA (cDNA) from skin of normal volunteers, as well as non-lesional and lesional skin from psoriatic patients, were used for deep sequencing of the β-chain and γ-chain, respectively. From these initial studies, we were able to fully characterize the TCR repertoire of each sample, and compare diseased and healthy skin both between and within an individual patient.

## Methods

### Skin samples

Normal skin biopsies from healthy volunteers (n=7), and non-lesional (n=5) and lesional skin (n=8) from patients with psoriasis, were obtained under Rockefeller University Institutional Review Board-approved protocols (IRB numbers MLO-0651 and JKR-0742), and stored in a cryovial in liquid nitrogen until use. Written informed consent was obtained and the study was performed in adherence with the Declaration of Helsinki. Data regarding patient demographics is found in Table S1. All psoriasis samples were obtained from patients with moderate-to-severe disease, which was determined by a psoriasis area severity index (PASI score) of more than 12 (moderate-to-severe psoriasis vulgaris with >10% body surface area involvement).

### Nucleic acid isolation

Frozen skin biopsies were processed in 1mL of RLT buffer (Qiagen, Venlo, Limburg) with 1% β-mercaptoethanol, through several rounds of homogenation (Polytron PT-10-35 GT-Kinematica) and sonication (Sonics Vibra cell; model VCX130). In brief, samples were homogenized for several 30-second intervals until the sample was significantly disrupted; samples were then sonicated for 6 pulses of 1–2 seconds (at an amplitude of 80–100) to fully lyse the cells. Genomic DNA and RNA was isolated using the Qiagen All-Prep DNA/RNA/Protein kit (Qiagen, Venlo, Limburg) (Catalog number 80004), following the manufacturer’s instructions. In brief, cell lysate was added to a DNA binding column and centrifuged. The flow-through containing RNA and protein was then added to a separate RNA binding column and centrifuged. Each column containing either the DNA or RNA from a sample was then washed and the appropriate nucleic acid was eluted, according to manufactures instructions.

### Deep sequencing

6–8μg of genomic DNA (at a concentration of 50ng/μl), was used for survey level deep sequencing of the β-chain, using the ImmunoSEQ platform (Adaptive Biotechnologies, Seattle, WA, USA). 2μg of RNA was used for reverse transcription using the High Capacity cDNA Reverse Transcriptase Kit (Applied Biosystems, Carlsbad, CA) (Catalog number 4368814), according to manufacturer’s protocols, and the entirety of the cDNA reaction was sent to Adaptive biotechnologies for survey level deep sequencing of the γ-chain using the ImmunoSEQ platform. Details of the deep sequencing assay are as follows: the TCRβ and TCRγ CD3 region was amplified and sequenced using the ImmunoSEQ assay (Adaptive Biotechnologies, Seattle, WA). In this assay, a multiplex PCR system was used to amplify the rearranged CDR3β and CDR3γ sequences from sample DNA or cDNA, respectively. The 87-base-pair fragment is sufficient to identify the VDJ region spanning each unique CDR3β. Amplicons were sequenced using the Illumina platform. TCRβ and TCRγ V, D and J gene definitions were provided by the IMGT database ( www.imgt.org). The assay is quantitative, having used a complete synthetic repertoire of TCRs to establish an amplification baseline and adjust the assay chemistry to correct for primer bias. In addition, barcoded, spiked-in synthetic templates were used to measure the degree of sequencing coverage and residual PCR bias. This information was used for further PCR bias correction and to estimate the abundance of sequenceable templates in each sample. The resulting data is filtered and clustered using both the relative frequency ratio between similar clones and a modified nearest-neighbor algorithm, to merge closely related sequences and remove both PCR and sequencing errors. Data was analyzed using the ImmunoSEQ analyzer toolset, as further described below.

### Data analysis

Sequencing data were analyzed using the immunoSEQ Analyzer ( https://clients.adaptivebiotech.com/login). Entropy H (i.e. Shannon’s Entropy), is a measure of the richness and uniformity of the TCR repertoire’s frequency distribution

$$H=-\sum_{i=1}^{N}P_{i}\log_{2}P_{i},$$

in which N is the number of unique clones and P _i_ is the frequency of clone i. Entropy ranges from 0 in a sample with only one clone, to H _max_ = log _2_N for a sample with a uniform distribution of clone frequencies. Monoclonal or oligoclonal samples have low entropy, and polyclonal highly diverse samples have entropy just under log _2_N. The measurement of entropy is sensitive to sampling depth, as sample size is the primary driver of the number of unique sequences in a sample. To account for variation in sequencing depth, entropy is normalized by its maximum value to give a metric that measure just sample evenness.

HN=HHmax.

Clonality ( Sherwood *et al.*, 2013) is defined as C = 1 - H _N_. Clonality equals 0 when all sequences are equally abundant, and equals 1 when a single sequence makes up the entire sample. Overlap scores ( Figure 3) excluded all paired non-lesional (NL) and lesional (LS) psoriatic skin comparisons were removed to prevent artificial inflation of the NL-LS comparison group. All data was analyzed using an unpaired students t-test or ANOVA, using the GraphPad Prism5 software. Error bars represent the standard error of the mean. P-values < 0.05 were considered significant.

## Results

Raw data for "Deep Sequencing of the T-cell Receptor Repertoire Demonstrates Polyclonal T-cell Infiltrates in Psoriasis" Click here for additional data file. Copyright: © 2015 Harden JL et al. 2015 Data associated with the article are available under the terms of the Creative Commons Zero "No rights reserved" data waiver (CC0 1.0 Public domain dedication).

### Deep sequencing demonstrates diverse and highly polyclonal αβ- and γδ- T-cell repertoires in psoriatic lesional skin

**Figure 1.**
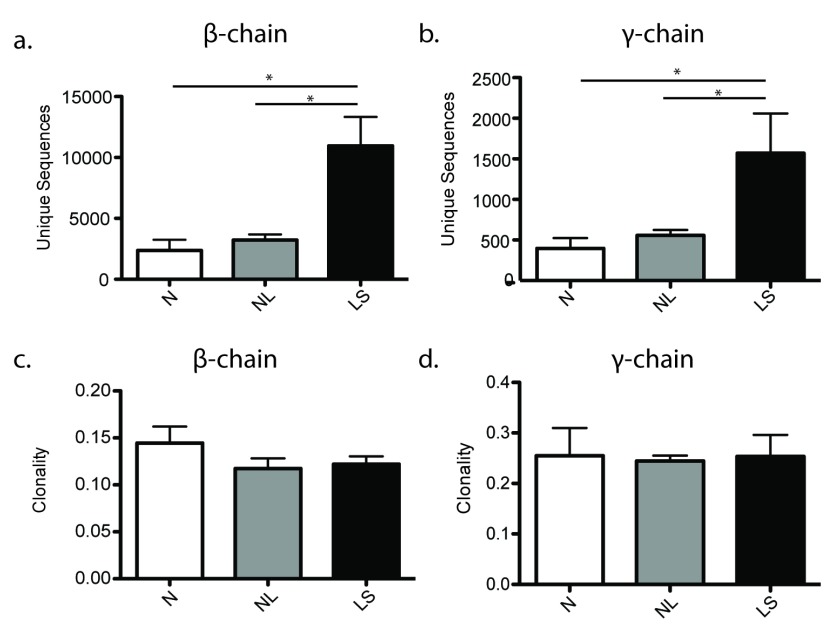
Psoriatic lesional skin has an increased number of unique TCRβ and TCRγ sequences and is not more clonal than normal and non-lesional skin. The total number of unique ( **a**) β- and ( **b**) γ-chain CDR3 sequences, and the clonality of the entire ( **c**) β- and ( **d**) γ-chain repertoire (y-axis) in normal and psoriatic lesional and non-lesional skin (x-axis). Error bars represent standard error of the mean. Statistics were performed using an unpaired t-test. * p < 0.05.

Deep sequencing identified an average of 2.7 × 10 ^3^ unique TCRβ-CDR3 (complementarity-determining region 3) sequences in normal skin, 3.2 × 10 ^3^ unique TCRβ-CDR3 in non-lesional skin, and an average of 10.9 × 10 ^3^ unique TCRβ-CDR3 sequences in psoriatic lesional skin, a significant three-fold increase compared to both normal (p = 0.0127) and non-lesional (p = 0.0295) skin ( Figure 1a). The number of unique sequences was also significantly increased (p = 0.0159) approximately three-fold for the γ-chain, with an average of 397 unique TCRγ-CDR3 sequences in normal skin, 558 unique TCRγ-CDR3 in non-lesional skin, and an average of 1.57 × 10 ^3^ unique TCRγ-CDR3 sequences in psoriatic lesional skin ( Figure 1b). The observation that there were more productive unique sequences in psoriatic skin is most likely a consequence of the increased numbers of T-cells well-known to occur in this disease ( Lowes *et al.*, 2014; Zaba *et al.*, 2007).

Clonality was used to compare the degree of clonal expansion across groups of samples. Psoriatic skin (both non-lesional and lesional) had slightly less clonality in the β-chain (i.e. more diverse and polyclonal population) than normal skin; however this finding was not statistically significant (p = 0.1255 for normal versus lesional; p = 0.1419 for non-lesional versus lesional) ( Figure 1c). The clonality of the γ-chain repertoire was very similar between normal, non-lesional and lesional psoriatic skin ( Figure 1d). Therefore, in combination with a clear signal of increased numbers of T-cells in psoriatic tissue, this suggests that the increase in T-cells is not due to the clonal expansion of a limited number of disease specific T-cells but the infiltration of large and diverse numbers of both αβ- and γδ- T-cells responding to an immune signaling cascade.

### Similar usage of variable (V) and joining (J) genes in both the β- and γ-chains of all samples

The variable (V) and joining (J) gene usage of both the β- and γ-chains was compared across the normal, non-lesional, and lesional psoriatic skin groups ( Figure S1). Previous studies have suggested significant variations in V- and J- gene usage between normal and psoriatic skin. Although we found trends supporting these previous observations, such as TCRVβ02.1 preferentially utilized in normal skin ( Chang *et al.*, 1995; Menssen *et al.*, 1995), much larger sample sizes would be required to verify potential gene usage differences.

### Clones in lesional skin are found in paired non-lesional skin at similar percentages

**Figure 2.**
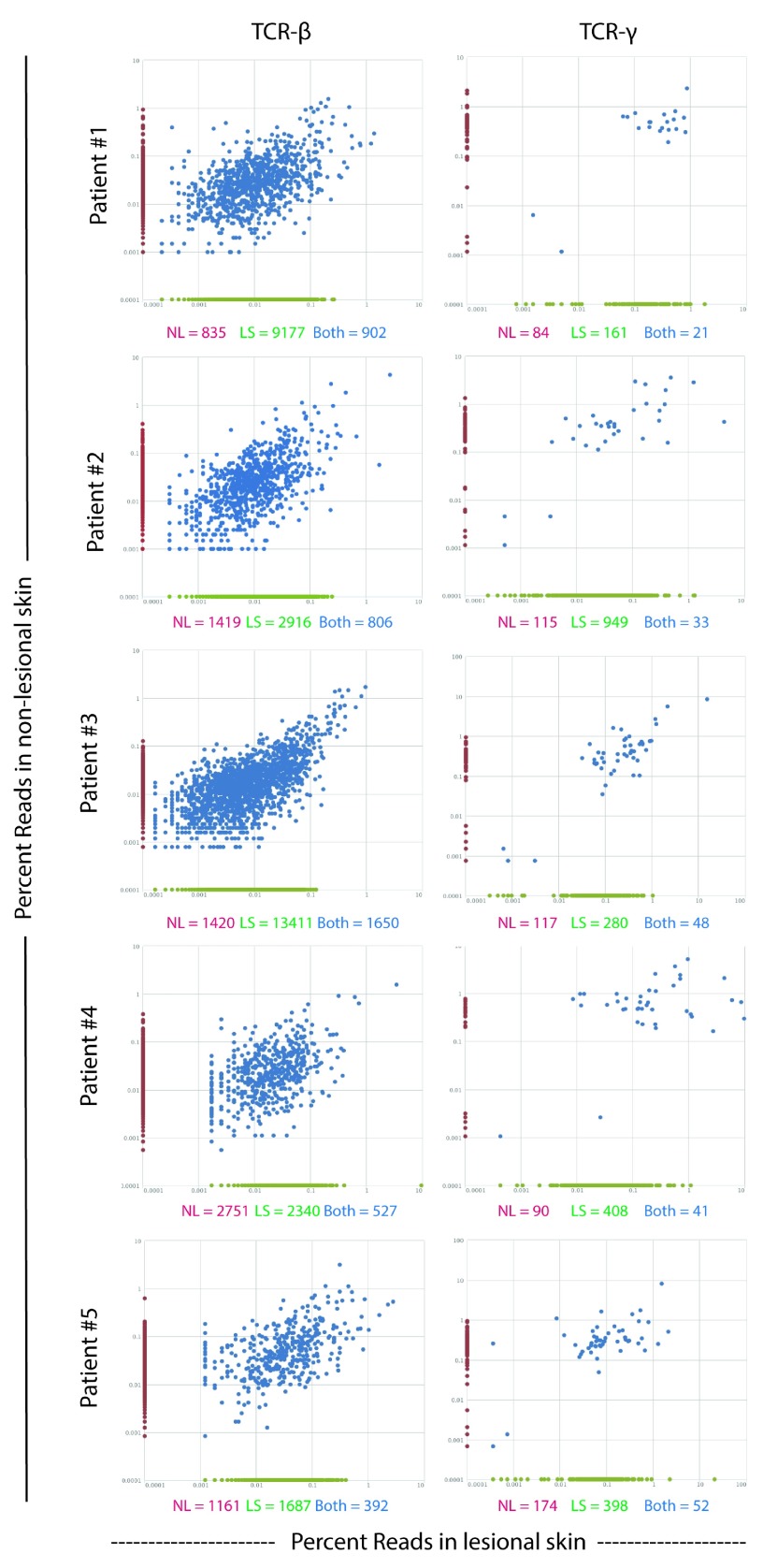
Common clones maintain similar frequency between non-lesional and lesional skin. Comparison of TCR **(left)** β- and **(right)** γ-chain CDR3 sequences in paired non-lesional and lesional psoriasis samples. Each dot represents one clone and the percent of total reads of a given clone in non-lesional (y-axis) and lesional (x-axis) skin are shown. Clones uniquely in non-lesional skin are located on the y-axis in red; clones uniquely in lesional skin are located on the x-axis in green; and clones found in both non-lesional and lesional skin are in blue. The absolute number of sequences in only non-lesional (red), only lesional (green), or both (blue) are located under each graph.

To determine if the most abundant clones in lesional skin may expand from a rare subset in non-lesional skin, we compared the β- and γ-chain TCR repertoire in paired lesional and non-lesional samples ( Figure 2). Although there were many clones uniquely present in only non-lesional and lesional skin, in all patients the most abundant clones in lesional skin were also the most abundant in non-lesional skin. This suggests that the abundance of clones in lesional skin is not the result of the expansion of a rare-subset in non-lesional skin, but is rather a general expansion and/or influx of many clones.

### Common sequences are found in the γ-chain repertoire of psoriatic skin

**Figure 3.**
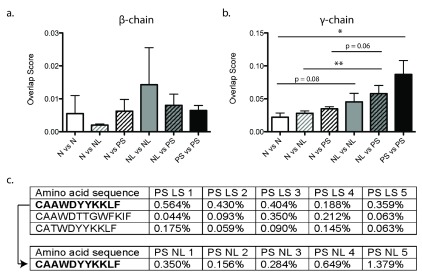
Increased sharing of TCRγ are found in psoriatic skin. Sequence similarity overlap scores (y-axis) obtained from the comparisons, normalized to total number of sequences and pooled into common groups (x-axis) for the ( **a**) β- and ( **b**) γ-chain. ( **c**) Common clones found within all lesional psoriatic skin samples (top), and one clone found in all psoriatic lesional and non-lesional skin (bottom). Error bars represent standard error of the mean. Statistics were performed using an unpaired t-test. * p < 0.05.

Although there is a highly diverse and polyclonal T-cell response in psoriasis, with no clear enrichment for particular variable (V) and joining (J) genes, there may still be clones specifically associated with psoriatic skin. If this is correct, it would be expected then that the degree of pair-wise sequence sharing would be greater within the psoriatic group. To test this hypothesis, the fraction of shared sequences between all pair-wise sets of samples was calculated ( Figure 3).

There was no significant difference (ANOVA p = 0.1114) in the overlap score for any comparisons of the β-chain ( Figure 3a). However, there were significant differences in overlap score comparisons for the γ-chain (ANOVA p = 0.0039) ( Figure 3b). The psoriatic lesional skin γ-chain repertoire exhibited significantly more pair-wise overlap than healthy controls. Additionally, the γ-chain repertoire of non-lesional skin compared to psoriatic skin contained significantly more overlap than comparison of non-lesional skin to normal skin. This finding suggests that unique populations of cutaneous γδ- T-cells may be present in psoriatic patients.

To further assess this hypothesis, we explored if there were any common TCRγ sequences between all of the samples within a group. There were no common γ-chain sequences among all the normal samples. However, there were 3 common γ-chain sequences found among all psoriatic lesional skin samples ( Figure 3c). Additionally, one of those γ-chain sequences was also found in all non-lesional skin samples as well. Regarding the β-chain, no common sequences were found in any group.

## Discussion

We have utilized the recent advances in next-generation sequencing to provide evidence for diverse and polyclonal αβ- and γδ- T-cell populations in psoriasis lesional skin. Although several previous studies have focused on the clonality within a range of Vβ- usages ( Chang *et al.*, 1995; Menssen *et al.*, 1995; Vollmer *et al.*, 2001), this is the first time sufficient numbers of sequences have been obtained to fully characterize the diversity of the TCR repertoire in psoriasis. We found that although lesional skin contained significantly more unique β- and γ- chain sequences than normal and non-lesional skin, lesional skin was highly polyclonal with no dominant T-cell clones. This finding supports a diverse and non-specific polyclonal T-cell infiltrate in psoriasis lesional skin.

Based on previous work, it may have been anticipated that there would be higher clonality compared to control samples, due to expansion of T-cells to an unknown antigen. The first gene associated with psoriasis (located at *PSORS1*) is HLA-Cw6 ( Perera *et al.*, 2012); it was thought that HLA-Cw6 likely predisposes to psoriasis by presentation of an unknown autoantigen. Mutations in other immune-related genes have recently been found to impose psoriasis-susceptibility, such as *IL-36RN* and *CARD14* ( Jordan *et al.*, 2012; Marrakchi *et al.*, 2011); however the immunological activities of these latter genes are not solely at the adaptive immune level, and play important roles in general inflammation.

Several previous studies utilizing TCR-PCR technology found the same clones present in lesional skin of a patient over time ( Chang *et al.*, 1995; Menssen *et al.*, 1995; Vollmer *et al.*, 2001), and these clones were absent in non-lesional skin. In our study, we found that the most prevalent clones in lesional skin were present at a similar amount (percent of reads) in non-lesional skin. Our data suggests that it is not an expansion of common non-lesional/lesional clone as the driver of psoriasis, but rather a general polyclonal T-cell expansion in psoriatic lesional skin.

Although αβ-T-cells are more prevalent than γδ T-cells in human skin ( Elbe *et al.*, 1996), it has recently been appreciated that γδ T-cells may contribute to psoriatic inflammation, as they can be major producers of IL-17, a key cytokine in psoriasis pathogenesis ( Cai *et al.*, 2012). We found minimal similarities between the TCRβ-repertoire of different patient samples despite the much greater sample sizes of TCRβ clones. However, three common TCRγ clones were found in all lesional skin samples, and one clone was found in all non-lesional and lesional skin, but was absent in normal skin. This finding merits further investigation with larger samples sizes to determine if particular γδ- T-cells are common among psoriasis patients and may represent a population(s) responding to a similar antigen(s).

In conclusion, we have provided the first deep sequencing results of the entire β- and γ- T-cell repertoire in normal, non-lesional, and lesional human skin. Our findings demonstrate highly polyclonal αβ- and γδ- T-cell populations in psoriasis lesional skin, with the most common clones being present in both non-lesional and lesional skin. Lastly, there may be possible contributions of specific γδ- T-cell in psoriasis, as evidenced by common CDR3 sequences between patients.

## Data availability

The data referenced by this article are under copyright with the following copyright statement: Copyright: © 2015 Harden JL et al.

Data associated with the article are available under the terms of the Creative Commons Zero "No rights reserved" data waiver (CC0 1.0 Public domain dedication). 

*F1000Research*: Dataset 1. Raw data for "Deep Sequencing of the T-cell Receptor Repertoire Demonstrates Polyclonal T-cell Infiltrates in Psoriasis", 10.5256/f1000research.6756.d97231 ( Harden *et al.*, 2015).

This data is also available from the Adaptive Biotechnology ImmunoSEQ site ( http://adaptivebiotech.com/pub/Harden-2015-F1000Res) which provides access to ImmunoSEQ Analyzer and other tools used for analyses.

## Supplementary material

Supplementary material for "Deep Sequencing of the T-cell Receptor Repertoire Demonstrates Polyclonal T-cell Infiltrates in Psoriasis".
