# Supplementary material for: Deep Sequencing of the T-cell Receptor Repertoire Demonstrates Polyclonal T-cell Infiltrates in Psoriasis
Source: F1000Res. 2015 Aug 3;4:460. [Version 1] doi: 10.12688/f1000research.6756.1 (PMC4648215; doi:10.12688/f1000research.6756.1)
Supplement: Supplementary file 2 [file f1000research-4-7258-s0001.tgz › a41083da-8299-4845-a60b-31bdc21bb91b.pdf]

Table S1 – Patient demographics and study information

Sample	Disease Severity (PASI)	Gender	Age (y)	Ethnicity	gDNA (β-chain sequencing)	cDNA (γ-chain sequencing)
Normal 1	n/a	M	41	Black	Y	N
Normal 2	n/a	M	48	Unidentified	Y	N
Normal 3	n/a	M	52	Black	Y	Y
Normal 4	n/a	F	46	Black	Y	Y
Normal 5	n/a	M	55	Black	Y	Y
Normal 6	n/a	M	43	Black	N	Y
Normal 7	n/a	M	40	Black	N	Y
Non-lesional 1	Moderate-severe (17.8)	F	57	Hispanic/Latino	Y	Y
Non-lesional 2	Moderate-severe (26)	M	42	Hispanic/Latino	Y	Y
Non-lesional 3	Moderate-severe (21)	F	62	White	Y	Y
Non-lesional 4	Moderate-severe (32.7)	M	44	Hispanic/Latino	Y	Y
Non-lesional 5	Moderate-severe (12)	M	23	White	Y	Y
Lesional 1	Moderate-severe (17.8)	F	57	Hispanic/Latino	Y	Y
Lesional 2	Moderate-severe (26)	M	42	Hispanic/Latino	Y	Y
Lesional 3	Moderate-severe (21)	F	62	White	Y	Y
Lesional 4	Moderate-severe (32.7)	M	44	Hispanic/Latino	Y	Y
Lesional 5	Moderate-severe (12)	M	23	White	Y	Y
Lesional 6	Moderate-severe (20.5)	M	69	White	Y	N
Lesional 7	Moderate-severe (n/a)	M	63	Asian	Y	N
Lesional 8	Moderate-severe (n/a)	M	25	Black	Y	N

Figure S1

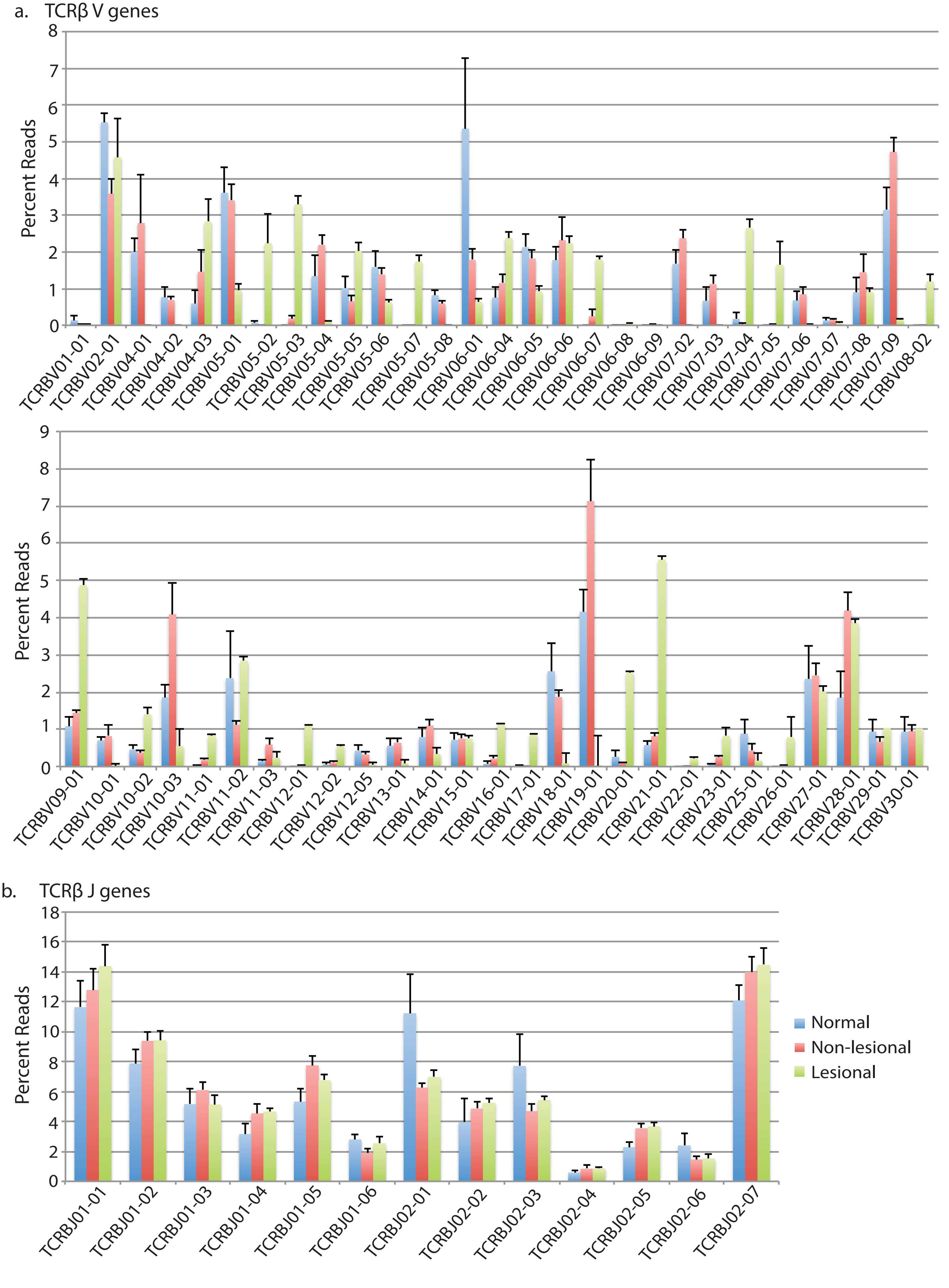
a. TCRβ V genes
Percent Reads
TCRBV01-01
TCRBV02-01
TCRBV04-01
TCRBV04-02
TCRBV04-03
TCRBV05-01
TCRBV05-02
TCRBV05-03
TCRBV05-04
TCRBV05-05
TCRBV05-06
TCRBV05-07
TCRBV05-08
TCRBV06-01
TCRBV06-04
TCRBV06-05
TCRBV06-06
TCRBV06-07
TCRBV06-08
TCRBV06-09
TCRBV07-02
TCRBV07-03
TCRBV07-04
TCRBV07-05
TCRBV07-06
TCRBV07-07
TCRBV07-08
TCRBV07-09
TCRBV08-02
TCRBV09-01
TCRBV10-01
TCRBV10-02
TCRBV10-03
TCRBV11-01
TCRBV11-02
TCRBV11-03
TCRBV12-01
TCRBV12-02
TCRBV12-05
TCRBV13-01
TCRBV14-01
TCRBV15-01
TCRBV16-01
TCRBV17-01
TCRBV18-01
TCRBV19-01
TCRBV20-01
TCRBV21-01
TCRBV22-01
TCRBV23-01
TCRBV25-01
TCRBV26-01
TCRBV27-01
TCRBV28-01
TCRBV29-01
TCRBV30-01
b. TCRβ J genes
TCRBJ01-01
TCRBJ01-02
TCRBJ01-03
TCRBJ01-04
TCRBJ01-05
TCRBJ01-06
TCRBJ02-01
TCRBJ02-02
TCRBJ02-03
TCRBJ02-04
TCRBJ02-05
TCRBJ02-06
TCRBJ02-07
Normal
Non-lesional
Lesional

Figure S1 continued

c. TCRγ V genes

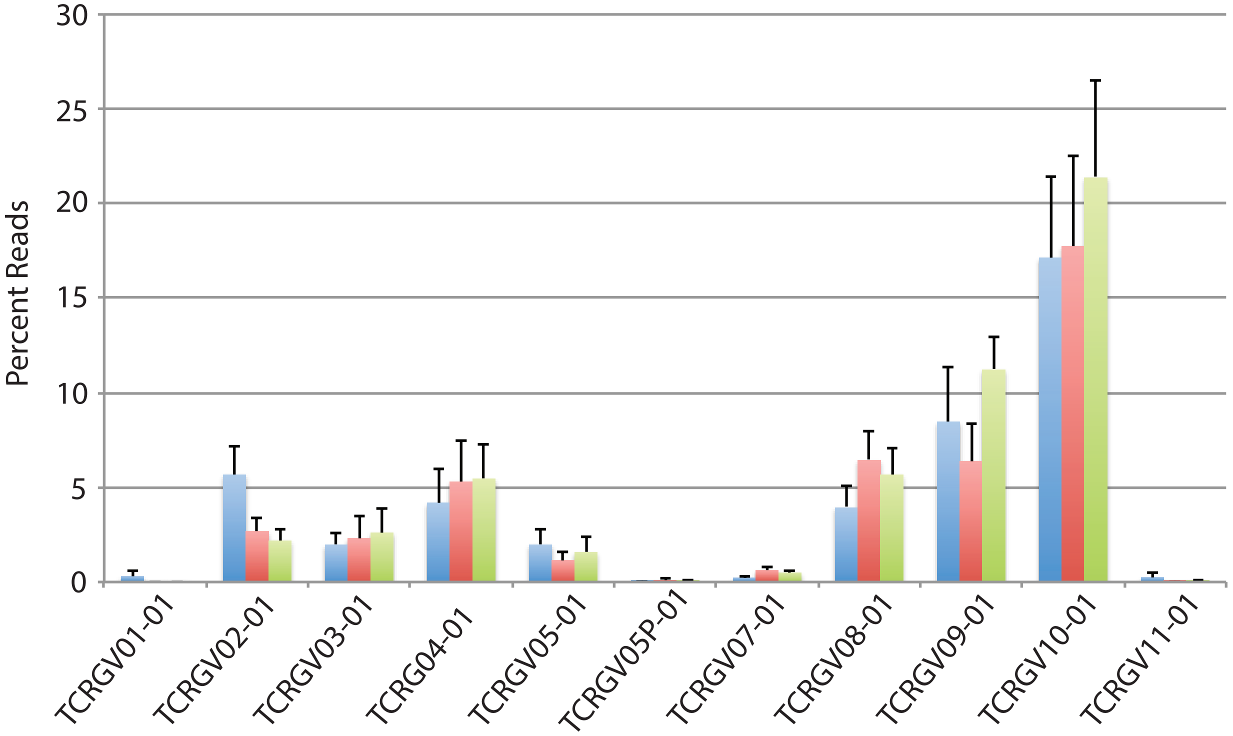

d. TCRγ J genes

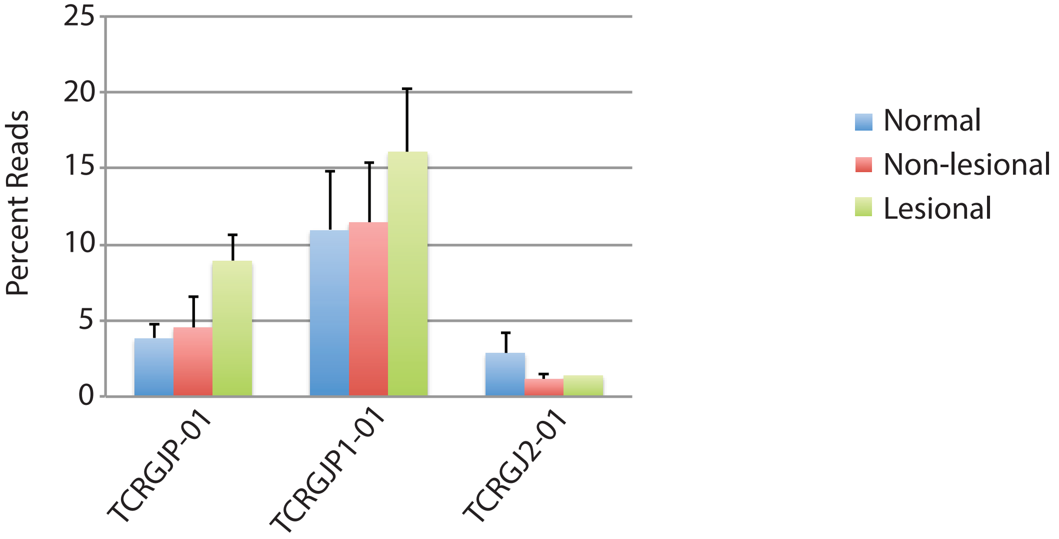

Figure S1: The expression of all TCRγ and TCRβ V- and J- genes in normal, non-lesional and lesional skin. The percent of reads of a gene in normal (blue), non-lesional (red), or lesional (green) skin is shown for (a) TCRβ-V genes, (b) TCRβ-J genes, (c) TCRγ-V genes, and **(d)** TCRγ-J genes. n = 5 for normal, n = 5 for non-lesional, and n = 8 for lesional. Error bars = S.E.M.
