# Supplementary material for: Deep Sequencing of the T-cell Receptor Repertoire Demonstrates Polyclonal T-cell Infiltrates in Psoriasis
Source: F1000Res. 2015 Aug 3;4:460. [Version 1] doi: 10.12688/f1000research.6756.1 (PMC4648215; doi:10.12688/f1000research.6756.1)
Supplement: Peer review history

# Peer review: Referee response for version 1

**DOI:** 10.5256/f1000research.7258.r10142

**Published:** 2015-09-09

**Authors:** Jun Yan, Yihua Cai

**Affiliations:** 1 Department of Microbiology and Immunology, School of Medicine, University of Louisville, Louisville, KY, USA

The manuscript by Harden *et al. *entitled “Deep Sequencing of the T cell Receptor Repertoire Demonstrates Polyclonal T-cell Infiltrates in Psoriasis” describes the results of high-throughput deep sequencing of the TCR beta and gamma chains in normal healthy skin and psoriatic lesional and nonlesional skin tissues. The authors found that although there is a significant increase in the abundance of unique beta and gamma TCR sequences in poriatic lesional skin, the entire T cell repertoire in psoriasis is polyclonal. More strikingly, they found three common sequences of gamma chain repertoire in psoriatic skin. These are really exciting findings and provide novel insights into understanding psoriasis immunopathogenesis.

Minor comments:

1. The rationale for using genomic DNA (gDNA) for TCR beta chain while cDNA for TCR gamma chain sequencing needs to be clearly delineated. This is particularly important given the fact that different target enrichment solutions have its own pros and cons.
2. The authors conclude that the entire T cell repertoire in psoriasis is rather polyclonal with similar diversity in normal vs non-lesional skin, which is contradictory to previous reports. The authors may want to discuss this further, e.g. whether this discrepancy is due to different technology used or other factors.
3. Were psoriatic lesional and non-lesional skin tissues paired?

We have read this submission. We believe that we have an appropriate level of expertise to confirm that it is of an acceptable scientific standard.

# Peer review: Referee response for version 1

**DOI:** 10.5256/f1000research.7258.r10141

**Published:** 2015-09-08

**Authors:** Liv Eidsmo, Stanley Cheuk

**Affiliations:** 1 Unit of Dermatology and Venereology, Department of Medicine, Karolinska Institutet, Solna, Sweden

Harden *et al.*, provide the first deep sequence analysis of the T cell receptor in lesional and non-lesional skin collected from psoriasis patients. The authors simultaneously track and quantify of the V-β (from DNA) and V-γ (from RNA) T cell receptor within the same skin tissue samples. The data suggest that the T cell infiltrate in full-thickness psoriasis lesions is clonally diverse and the authors could not reproduce previous results showing oligoclonal expansion of TCR ab within the skin.The authors do however suggest clonal enrichment of TCR γ in psoriasis patients, both in lesional and non-lesional skin, in comparison to healthy skin. The manuscript is straightforward but there are several points that the authors could comment or discuss further as listed below.

**Major concerns:**

1. The majority of infiltrating T cells in psoriasis are found in the dermal compartment of the skin with only a small population of T cells infiltrating epidermis. However, an enrichment of pathogenic IL-17 and IL-22 producing T cells in epidermis as compared to dermis of psoriasis has been shown in a number of reports ( Res *et al.*, 2010; Hijnen *et al.*, 2013; Cheuk *et al.*, 2014). Of interest, the oligoclonal T cell expansion shown by Menssen *et al*. (1995) in the same psoriasis plaques over time were detected in epidermis and intraepithelial oligoclonal T cell expansion was also suggested by Lin *et al. *(2001). Without dissecting epidermis and dermis, it is difficult to draw the conclusion that psoriasis is a “highly polyclonal” response lacking oligo-clonal expansion.
2. Intraindividual comparison of two lesional and two non-lesional samples would be interesting to determine the heterogeneity of T cell infiltration in different sites of inflamed and non-affected skin.
3. Please comment on the skewed ethnicity between the healthy controls and the patient group analyzed. Could this affect the interpretation of the γδ data, in which the authors showed overlap among NL and LS as compared to the controls?
4. Did quantification of TCRg analysed in RNA or DNA give identical results or was a bias in the quantification introduced when analysing cDNA?
5. Figure 3: How the overlap score is calculated is unclear. Is that equivalent to “ the fraction of shared sequences between all pair-wise sets of samples”?  A more detail description will be appreciated.

 

**Minor points:**

- Abstract row 14: “...these close were often...” should probably read “...these clones were often...”.
- Figure 2: Out of 5 donors, 2 (or 3) did not show much difference in the total number of unique sequences between NL and LS. That was inconsistent with data from figure 1. Please comment on that.
- The sharing of specific TCR γ clones among different patients but absent in healthy controls is intriguing. Are these clones consistent with the infiltrating gd T cells in psoriasis described by Laggner *et al. *(2011)
- Figure 3: Were the pair-wised comparison from the same donor omitted or included in the calculation since some of the LS and NL were collected from the same donor? That would affect the interpretation statistical testing between the groups concerning the healthy control and the groups concerning only psoriasis patients. Please comment on that.
- Figure 3: The overlapping score or fraction of shared sequences between different donors in the case of TCR-β is difficult to interpret since the donor HLA type is not shown.

We have read this submission. We believe that we have an appropriate level of expertise to confirm that it is of an acceptable scientific standard.
